# How far do adult turtles move? Home range and dispersal of *Kinosternon integrum*

**DOI:** 10.1002/ece3.3339

**Published:** 2017-09-07

**Authors:** Ailed Pérez‐Pérez, Ana Esthela López‐Moreno, Orlando Suárez‐Rodríguez, Justin Lloyd Rheubert, Oswaldo Hernández‐Gallegos

**Affiliations:** ^1^ Laboratorio de Herpetología Facultad de Ciencias Universidad Autónoma del Estado de México Toluca Estado de México Mexico; ^2^ College of Sciences The University of Findlay Findlay OH USA

**Keywords:** activity, freshwater turtles, kernel density, México, minimum convex polygons, movement patterns, telemetry

## Abstract

We describe the home range and movements of a population of *Kinosternon integrum* in Tonatico, Estado de México, México, over 3.5 years (during rainy and dry season months) using radiotelemetry in 37 adult turtles. The results showed that the home range of *K. integrum* was 0.151 ± 0.051 ha using 50% kernel density estimator (KDE), and 0.657 ± 0.214 ha using 95% KDE; the home range did not vary between sexes. *Kinosternon integrum* showed low distances traveled 51.44 ± 4.50 m, where 87.3% (*n* = 373) of movements were <100 m. The distance traveled differed by season, and movement category (aquatic and terrestrial movements). The shortest distance occurred during the dry season, during which some individuals move to estivation sites, and these movements were shorter than movements to artificial ponds (cattle ponds). In this population, home range and movement are similar to other species of the genus *Kinosternon*. Overall, the results indicate than *K. integrum* are highly dependent on aquatic habitats, but also utilize the terrestrial habitats for different biological activities, and to maintain viable populations. Therefore, the conservation of the entire inhabited area is fundamental. This study highlights the need to increase the studies, in Central México, concerning habitat use of freshwater turtles in order to increase the efficiency of conservation strategies.

## 1 INTRODUCTION

In turtles, knowledge about how individuals move within their habitat is essential to understanding the ecological rationale of dispersion, migrations, and movements to other habitats (Gibbons, Greene, & Congdon, 1990). Within turtles in general, high variation in home ranges and movements have been documented both inter‐ and intraspecifically (Gibbons et al., 1990; Slavenko, Itescu, Ihlow, & Meiri, 2016). Different extrinsic factors have been identified that influence the movements of reptiles that inhabit freshwater systems including the following: periodic droughts, weather, season, size of wetlands, environmental variation, and distribution of resources (Bennett, Gibbons, & Franson, 1970; McIntyre & Wiens, 1999; Milam & Melvin, 2001; Plummer, Mills, & Allen, 1997; Roe & Georges, 2008). However, intrinsic factors such as sex, size, sexual maturity, dominance, and physiological and behavioral traits (Gibbons et al., 1990; Hall & Steidl, 2007; Litzgus & Mousseau, 2004; Morreale, Gibbons, & Congdon, 1984; Roe & Georges, 2008; Stone, 2001; Swingland, 1983) can also influence the home range and movement patterns.

Some species of freshwater turtles exhibit high fidelity for their habitats (Cagle, 1944); in particular, turtles of the family Kinosternidae present small home ranges and low mobility compared with other freshwater turtles (Cordero & Swarth, 2010; Ennen & Scott, 2008; Slavenko et al., 2016). Nevertheless, some individuals of the populations are prone to long‐distance movements, perhaps to explore available habitat and resources (Hall & Steidl, 2007; Powell, 2000; Smar & Chambers, 2005). In freshwater turtles, movements depend strongly on the access to water in their habitat (Hall & Steidl, 2007; Stone, 2001). Water systems are highly variable environments and conditions can change dramatically between different ponds or within the same pond over time (Euliss et al., 2004), especially in temporary ponds, which occasionally dry (Bauder, 2005; Kennett & Georges, 1990).

The quality and quantity of resources varies temporally and spatially, and variation in the use of resources can influence the movement patterns and space use by animals (Roe & Georges, 2007). In habitats where extremely high temperatures or drought conditions occur during any season of the year, resources such as water or food may be scarce (Ligon & Stone, 2003; Litzgus & Mousseau, 2004; Pinder, Storey, & Ultsch, 1992; Storey, 2001). Therefore, individuals have developed different strategies to survive (Hall & Steidl, 2007) such as a) migration toward permanent water bodies, b) congregation in the local aquatic habitat (Ligon & Stone, 2003), or c) estivation until the water supply is restored (Iverson, 1990; Ligon & Stone, 2003; Wygoda, 1979); the use of these strategies manifest the necessity of identifying variations in home range, movements, and habitat requirements of freshwater turtles in order to effectively design and implement conservation strategies (Pittman & Dorcas, 2009; Rizkalla & Swihart, 2006), as well as improve management plans of endemic species, especially those inhabiting highly urbanized and polluted areas (Harden, Price, & Dorcas, 2009).

**Figure 1:**
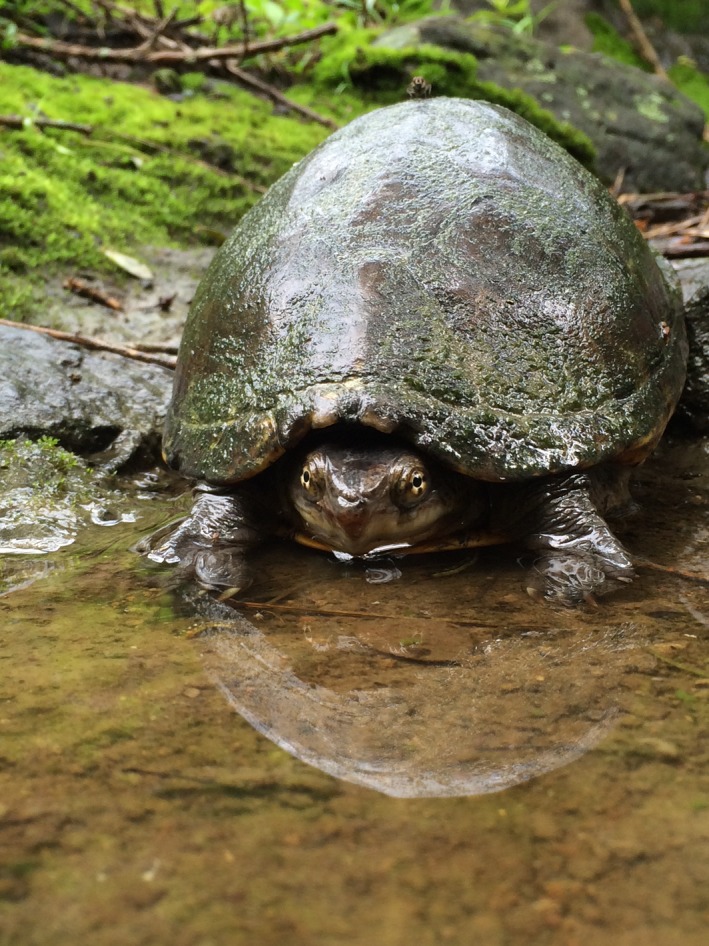
Adult female of *Kinosternon integrum* from Tonatico, Estado de México, México (Photograph by Ailed Pérez‐Pérez)

*Kinosternon integrum* is endemic to México (Lemos‐Espinal & Smith, 2009; Figure 1), and, although considered least concern by the IUCN Red List, is under special protection by Mexican laws (SEMARNAT, 2010). Vulnerability was assessed using an Environmental Vulnerability Score (EVS) ranking *K. integrum* an 11, placing it as a medium vulnerability species (Wilson, Mata‐Silva, & Johnson, 2013). Although it is the most widely distributed and the most commonly encountered freshwater turtle in México (Iverson, 1999), it is not excluded from the issues that affect freshwater turtles worldwide (e.g., water pollution, habitat degradation; Dudgeon et al., 2006). Currently, data concerning home range and movements in the genus *Kinosternon* in México are sparse and, to our knowledge, movement patterns have only been reported for one species in México (*Kinosternon leucostomum*; Morales‐Verdeja & Vogt, 1997). Therefore, the purpose of this study was to evaluate the home range of *K. integrum*, as well as the movements and their variations according to sex, season, and category (aquatic and terrestrial movements), across years and estimate which of these factors affect the movements. Finally, the home range and movements were compared with other species of family Kinosternidae.

## 2 MATERIALS AND METHODS

### 2.1 Study site

**Figure 2:**
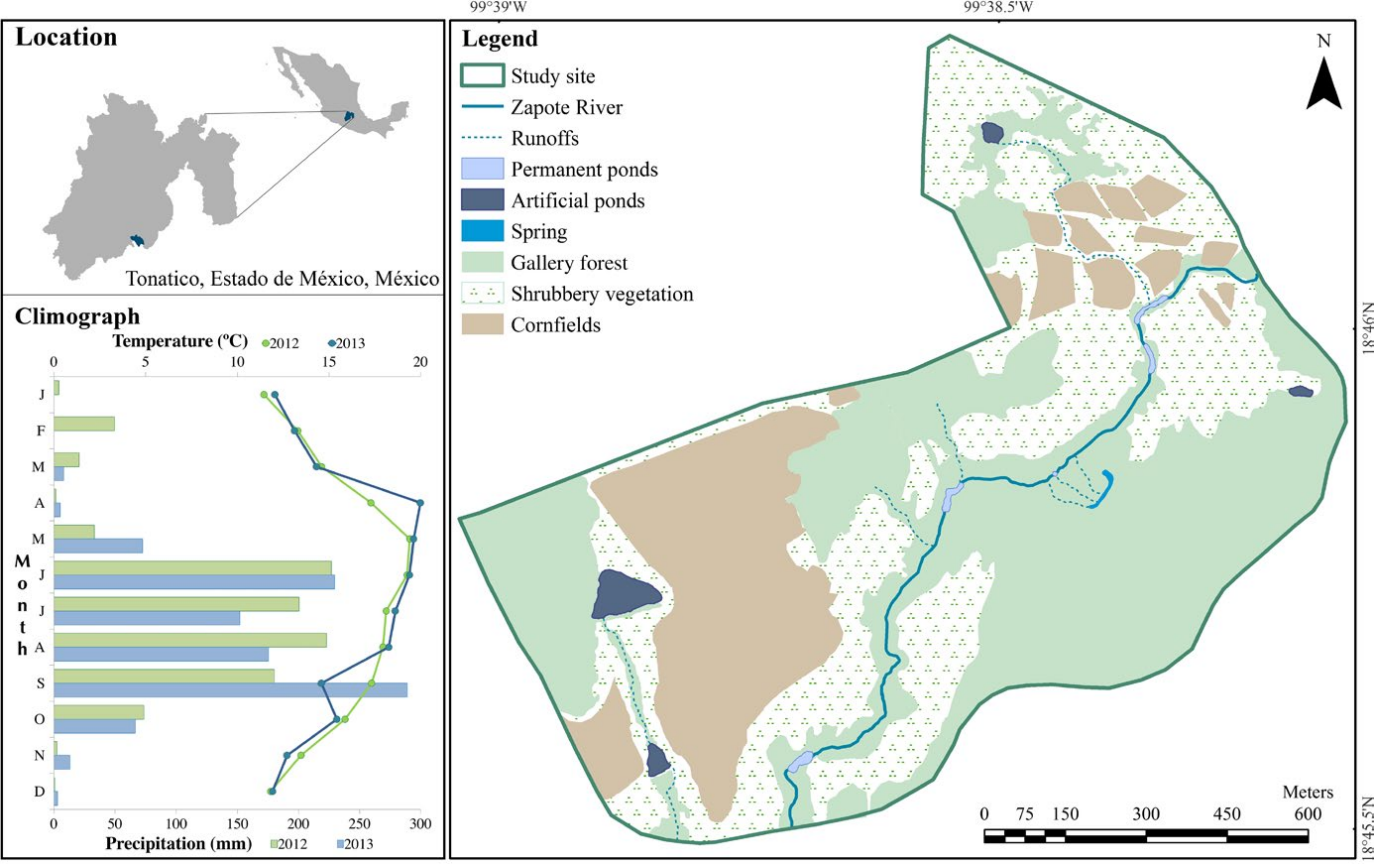
Study site and climograph (2012–2013) of Tonatico, Estado de México, México

This study was carried out in Zapote River, located in Tonatico, Estado de México, México (18°45′ N, 99°38′ W; 1,500 m.a.s.l. Figure 2). The area has a semiwarm humid climate with summer rains with average annual temperature ranging from 18 to 22°C, and annual precipitation ranging from 1,000 to 1,200 mm (INEGI, 2008; Luna, Morrone, & Espinosa, 2007). The rainy season typically occurs from mid‐June to mid‐September but sometimes is extended and includes the end of May and beginning of October, and the dry season occurs during the remaining months (Hernández‐Gallegos & Domínguez‐Vega, 2012; Figure 2). The Zapote River is fed by springs and runoffs, and the water level in the river changes considerably due to seasonal and annual variations in rainfall. Most of the river dries prior to the rainy season during April and May (the warmer and drier months of the year), and only a few permanent ponds remain (Figure 2). When the summer rains begin, the amount of water increases, and the water flow becomes continuous. Sand and rocks dominate the substratum, and the dominant vegetation in the riverbank is gallery forest (trees and shrubs).

### 2.2 Sampling methods

Samplings were performed from December 2012 to May 2016, and turtles were captured by hand and snorkeling along 1.25 km of the Zapote River. All turtles were sexed and weighed, and their straight carapace length and curved carapace length were measured. All turtles were marked using shell notching following Cagle (1939). Individuals measuring more than 120 mm of curved carapace length were considered adults (Brauer‐Robleda, 2009; Macip‐Ríos, 2005). Thirty‐seven adult turtles (18 females, 19 males; carapace length >120 mm; weight >300 g) were fitted with radiotransmitters (LL Electronics, Mahomet IL, model LF2 CR2477; weight 16.5 g, did not exceed 7% of body mass), which were attached to the posterior marginal scutes with silicon sealer. Turtles were held in the laboratory for 48 hr before being released at their exact capture locations. Turtles were monitored by a yagi antenna and a receiver (LL Electronics MN4000); the locations were recorded using a global positioning system unit (GPS, Garmin Oregon). Turtles were tracked at different periods throughout the sampling period (Table 1); the relocations were performed fortnightly; the sampling area of relocations included 1.12 km^2^ (Figure 2), due the movements of the turtles to other habitats (estivation sites, spring, artificial ponds, and runoffs). Movements from the river to other habitats (estivation sites, artificial ponds, and marsh) were considered terrestrial movements, whereas movements within the river and within artificial ponds were considered aquatic movements.

**Table 1:** Characteristics of tracking, home range, and movements of 37 adult turtles of *Kinosternon integrum* in Tonatico, Estado de México, México. Females (H), Males (M). ^❊^movements into the river, ^✣^movements to estivation sites, ^❖^movements to artificial ponds, and ^♦^movements to spring

Turtle ID	No. of Weeks tracked	No. of locations	Kernel (ha)		Area	Mean of distance (m)	Range of movements (m)	Date of tracked
			50%	95%	MCP (ha)			
H 4^❊✣^	174.6	48	0.080	0.281	2.99	70.9	0.0–327.2	01/13–05/16
H 6^❊^	119.6	31	0.035	0.173	0.176	20.7	0.0–70.3	03/13–06/15
H 7^❊^	25.2	8			0.207	43.9	0.0–249.1	03/13–09/13
H 9^❊^	36.5	9			0.029	12.3	1.5–25.8	10/13–06/14
H 10^❊^	90.1	3			0.037	157.2	43.3–271.1	06/14–01/15
H 11^❊❖^	90.6	8			1.049	100.5	0.0–303.6	06/14–01/15
H 13^❊^	23	3			0.066	45.2	40.9–49.6	05/13–10/13
H 15^❊✣❖^	116.4	15	0.385	1.730	2.386	101.3	0.0–343.7	05/13–07/15
H 19^❖^	27.6	3			0.202	236.0	40.8–431.1	07/14–10/14
H 21^❊^	29.4	6			0.117	30.8	3.9–53.2	04/14–11/14
H 22^❖^	22.3	3			0.007	331.2	0.0–662.4	07/14–09/14
H 28^✣❖^	61.4	16	0.099	0.494	0.696	67.7	0.0–425.7	05/15–05/16
H 29^❊^	54	22	0.015	0.058	0.076	20.5	2.5–58.0	04/15–04/16
H 34^❊^	51.6	19	0.022	0.129	0.371	26.6	1.1–135.3	04/15–04/16
H 36^❊^	5.1	3			0.003	14.7	2.5–26.8	04/15–05/15
H 37^❖^	12.7	4			0.225	243.7	10.2–568.3	05/15–06/15
H 41^❊^	17	7			0.019	10.5	0.0–26.4	12/15–04/16
H 45^❊^	20.6	9			0.095	21.7	0.0–76.1	11/15–04/16
M 1^❊✣❖^	57.9	21	0.255	1.273	3.808	59.4	0.0–509.2	01/13–02/14
M 3^❊^	14.6	8			0.060	25.9	2.5–86.0	01/13–04/13
M 5^❊^	87.6	17	0.006	0.028	0.030	10.8	0.0–53.4	02/13–09/14
M 12^❊✣^	159.6	16	0.407	1.630	2.507	91.2	0.0–387.2	05/13–05/16
M 14^❊^	60.6	9			0.049	33.3	5.3–62.8	01/14–12/14
M 17^❊♦^	92.6	20	0.013	0.071	0.400	24.1	0.0–97.1	09/13–06/15
M 20^❊✣❖^	104	30	0.469	1.878	14.969	91.5	0.0–425.4	04/14–04/16
M 24^❊^	20	3			0.034	62.1	30.6–93.6	05/13–09/13
M 25^❊^	100.1	13			1.167	50.7	3.2–234.0	09/14–05/16
M 26^❊❖^	23.4	11			1.785	107.6	0.0–428.8	04/15–09/15
M 27^❊^	15.7	9			0.659	38.0	0.0–105.7	03/15–06/15
M 30^❊^	16.1	9			0.049	21.2	7.0–52.1	04/15–07/15
M 31^❊♦^	23.4	4			0.305	98.5	7.5–256.8	04/15–09/15
M 33^❊^	25.4	12			1.013	81.4	4.9–203.0	04/15–09/15
M 35^❊^	51.6	22	0.036	0.136	0.156	18.2	1.1–63.2	04/15–04/16
M 42^❊^	17	7			0.007	6.1	1.1–11.3	12/15–04/16
M 43^❊^	28.6	9			0.302	50.6	8.1–124.8	09/15–04/16
M 44^❊^	27.6	12			0.070	13.4	0.0–86.3	10/15–04/16
M 46^❊^	34.6	13			0.033	10.2	0.0–33.9	09/15–05/16

In studies concerning movements and home range, independence is not biologically possible (Blundell, Maier, & Debevec, 2001); animals typically move in a nonrandom fashion and will return repeatedly to important areas (e.g., foraging sites, or locations that provide shelter or other important resources); this causes strong autocorrelation (Powell, 2000; de Solla, Bonduriansky, & Brooks, 1999; Swihart & Slade, 1985). de Solla et al. (1999) and Blundell et al. (2001) demonstrated that the kernel densities do not require serial independence of observations; the autocorrelation had no apparent effect on linear estimates of home ranges with any kernel method. Due to this, to estimate home range, we calculated 50% and 95% kernel density estimator (KDE) (only for turtles with more than 15 locations; 6 females and 6 males), using R software (R Development Core Team, R Foundation for Statistical Computing, Vienna, Austria). Most locations were highly clustered; thus, we used least‐squares cross‐validation for optimum bandwidth selection (Bowman, 1984). Kernel method is one of the least biased and most precise home estimator (Seaman, Griffith, & Powell, 1998; Worton, 1989); 50% KDE reflected core area of home range, where the high intensity of use of 95% indicated size of home range (Donaldson & Echternacht, 2005). We calculated the home ranges size of all animals with at least three captures or relocations, using 100% minimum convex polygon method (MCP), and estimate total distance of the movements between locations using the Xtool Pro extension in ArcGis 10.3 (Environmental Systems Research).

### 2.3 Statistical analyses

The size of home range (50% and 95% KDE) was compared separately by two‐way ANOVA using as a factor sex and mobility of the animals (two levels; animals that remain in the river all time and individuals that performed terrestrial movements) as cofactors. The correlations of the home range with straight carapace length and weight of turtles were analyzed by linear regression. The correlation of the days between captures with distance of the movements was tested by simple regression; the correlation of the distance of movements with weight and carapace length was tested by means of the Spearman's rank test.

The best analysis to evaluate distance of the movements can be a linear mixed effect model (LME), which includes individuals as a random factor, as has been performed in other reptile studies, including freshwater turtles (*Tiliqua rugosa,* Kerr & Bull, 2006; *Sternotherus odoratus,* Rowe, Lehr, McCarthy, & Converse, 2009; *Chrysemys picta marginata,* Rowe & Dalgarn, 2010; *Sternotherus minor peltifer*, Ennen & Scott, 2013). According to von Ende (1993) and Schafer and Graham (2002), this form of analysis accommodated temporal autocorrelation among seasons or years as well as missing data because of loss and replacement of individuals. Due to this, to analyze differences in distance of movements, a linear mixed effect model was used that included individual turtles as a random effect; fixed factors included sex, year, season, and category of movements (aquatic or terrestrial). A *Student′s t test* was used to evaluate differences between terrestrial movements (river/artificial ponds and river/estivation sites). Before analyses, variable distance of the movements was natural log‐transformed, to address assumptions of normality (Kolmogorov–Smirnov, *p* > .05). According to a Levene's test, 75% of the factors (sex, year, and season) were homoscedastic (*p* > .05), only 25% (category of the movements) were not homoscedastic (*p* < .001). All statistics were performed in SPSS version 20.0, means were presented ± 1 standard error (unless otherwise noted) and results were deemed significant if *p *<* *.05.

## 3 RESULTS

### 3.1 Home range of *Kinosternon integrum*

The average tracking time was 52.3 ± 7.04 weeks (range 5.1–174.6 weeks; Table 1); the mean of number of relocations was 12.5 ± 1.5 per turtle (range 3–48 relocations; Table 1). The home range size for *K. integrum* for 50% KDE was 0.151 ± 0.051 ha and for 95% KDE was 0.657 ± 0.214 ha (Table 1). Home range did not differ between sexes (50% KDE, *F *= 0.279, *p *=* *.612; 95% KDE, *F *= 0.207, *p *=* *.662). The mobility of turtles affects the home range, turtles that remain in the river have a smaller home range than turtles that perform terrestrial movements (50% KDE, *F *=* *5.68, *p *<* *.05; 95% KDE, *F *= 6.08, *p *=* *.039); and in both cases, there is not an interaction of the Sex × Mobility (50% KDE, *F *=* *0.315, *p *=* *.590; 95% KDE, *F *= 0.309, *p *=* *.593). The home range size (95% KDE) was not correlated with weight (*R* ^*2*^ **= 18.44, *p *=* *.164) or straight carapace length (*R* ^*2*^ **= 0.008, *p *=* *.983). The mean of home range using MCP (37 turtles) was 0.977 ± 0.418 ha; in this study, we report the home range of *K. integrum* by MCP (Table 1), for comparisons to other turtles; however, MCP was not used in statistical analyses and comparisons between MCP and KDE were not performed.

### 3.2 Distance of the movements of *Kinosternon integrum*

The mean distance traveled by turtles was 51.44 ± 4.50 m (range 0–662.4 m), and 87.3% (*n* = 373) of movements were <100 m. Of the 37 turtles tracked, 13 individuals (35.1%) performed terrestrial movements at some point during the study (which represented 15.2% of the movements). The distance traveled by turtles was weakly correlated with the number of days between recaptures (*R* ^*2*^ **= 0.148, *p *<* *.001). The mean distance traveled was not correlated to straight length of carapace (*R*_*s*_ **= −0.121, *p *=* *.663), or weight (*R*_*s*_ **= −0.2759, *p *=* *.320).

**Figure 3:**
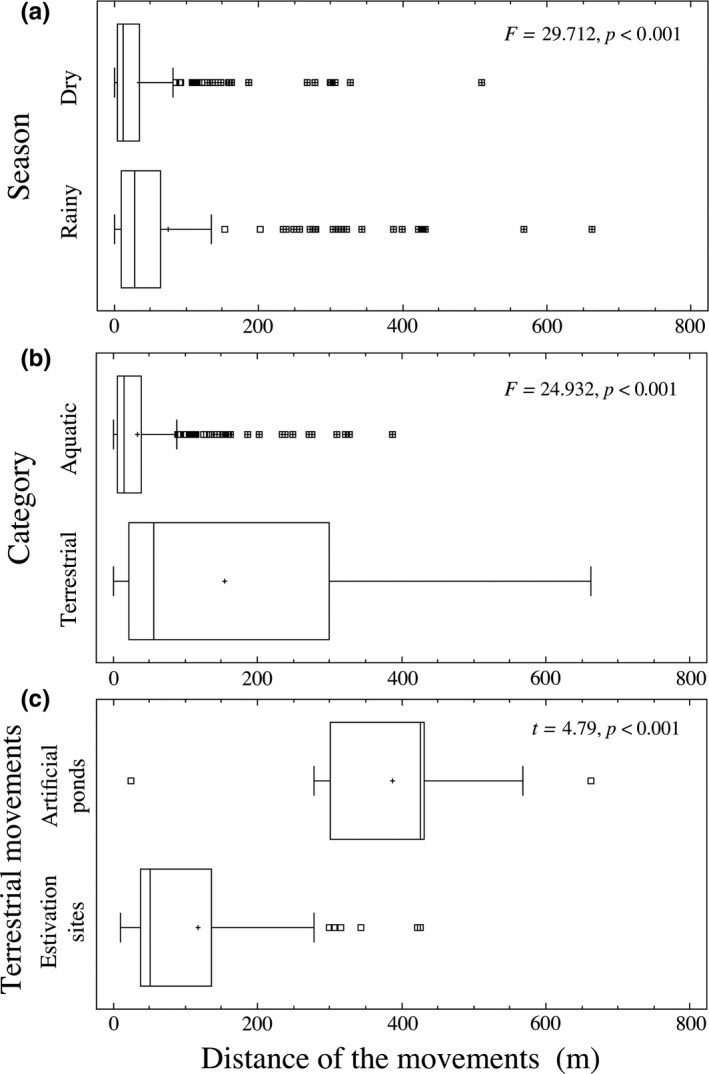
Comparison of the distance traveled by *Kinosternon integrum* between: a) Seasons, b) Category, and c) Terrestrial movements in Tonatico, Estado de México, México

**Figure 4:**
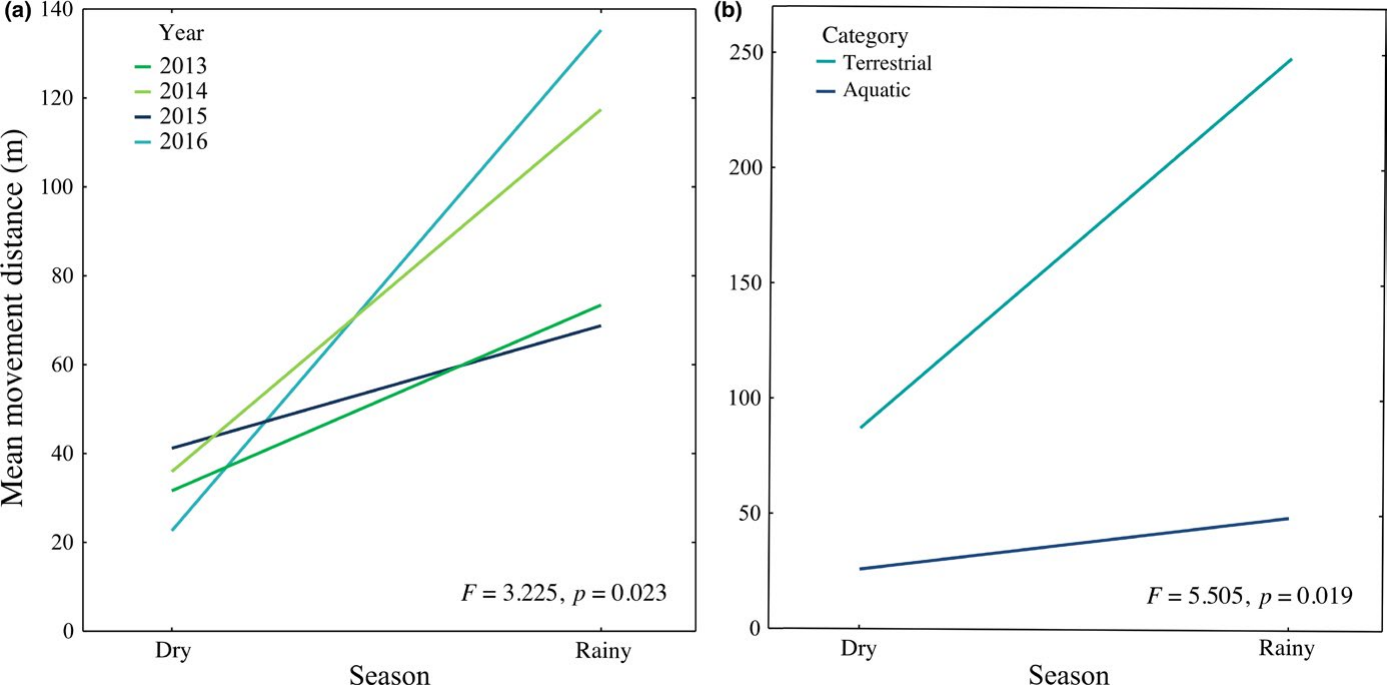
Mean movement distance of *Kinosternon integrum* according to: a) interaction between Year and Season, and b) interaction between Category and Season

**Figure 5:**
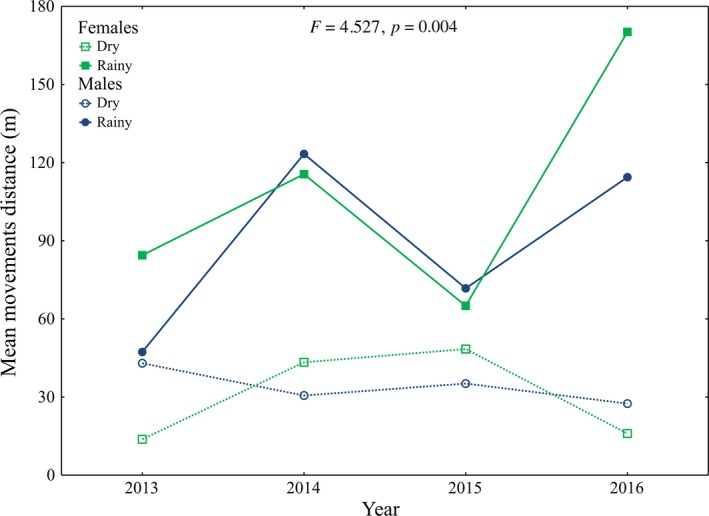
Mean of distance of movements of *Kinosternon integrum* according to three‐way interaction among Sex, Year, and Season

The distance traveled by turtles was mainly affected by season and by movement category (Tables 2 and 3). The movements during the rainy season were greater than the dry season (Table 3, Figure 3a), and terrestrial movements were greater than aquatic movements (Table 3, Figure 3b). When turtles moved from the river to artificial ponds (and return to the river) they traveled greater distances than those traveling to/from estivation sites (Table 3; Figure 3c). Only the interactions Year × Season, Season × Category, and Sex × Year × Season have an affect on the distance of the movements (Table 2). Differences in mean movement distance among years (Figure 4a) or category (Figure 4b) are not independent of the season. Moreover, differences in mean movement distance between sexes are not independent of the season or year (Figure 5).

**Table 2:** Results of linear mixed effects analyses for movement distance (log nat) of *Kinosternon integrum* in Tonatico, Estado de México, México, with Sex, Year, Season, and Category as fixed variables

Source	*df*	*F*	*p*
Sex	1	0.737	.397
Year	3	0.445	.721
Season	1	29.712	<.001^a^
Category	1	24.932	<.001^a^
Sex × Year	3	0.792	.500
Sex × Season	1	2.415	.121
Sex × Category	1	0.806	.370
Year × Season	3	3.225	.023^a^
Year × Category	3	0.603	.614
Category × Season	1	5.505	.019^a^
Sex × Year × Season	3	4.527	.004^a^
Sex × Year × Category	1	2.461	.087
Sex × Season × Category	1	2.828	.093
Year × Season × Category	2	0.546	.580
Sex × Year × Season × Category	1	0.927	.336

a Indicates significant test differences.

**Table 3:** Distance (m) moved by *Kinosternon integrum* according to Sex, Season, Year, Category, and Terrestrial movements in Tonatico, Estado de México, México

	Sex		Season^a^		Year				Category^a^		Terrestrial movements^a^	
	Females	Males	Dry	Rainy	2013	2014	2015	2016	Aquatic	Terrestrial	To/from artificial ponds	To/from estivation sites
Mean	56.1	47.93	35.07	74.33	45.55	61.89	54.81	36.06	34.11	154.71	387.48	117.81
*SE*	7.2	10.94	3.97	8.93	8.89	11.78	6.82	9.17	2.81	22.22	43.85	21.84
Range	0–662.4	0–509.2	0–509.2	0–622.4	0–509.2	0–662.4	0–568.3	1–425.7	0–387.2	1–662.4	24.4–662.4	10–425.7
*n*	200	227	247	180	81	91	188	67	365	62	13	34

a *p *<* *.001.

## 4 DISCUSSION

The movements of *K. integrum* in Tonatico, Estado de México, México, depend remarkably on the seasonality, especially rainfall. This population has high individual variation in the movements and did not exhibit a single movement pattern; while some individuals remain in the river continuously, others carry out seasonal migrations.

According to our results, the size of the home range in *K. integrum* was relatively large in respect to other species within the Kinosternidae family. However, compared with other species of freshwater turtles (Table 4), members of the Kinosternidae family have smaller home range sizes (Slavenko et al., 2016). Slavenko et al. (2016) reported that *Sternotherus odoratus* (a highly aquatic kinosternid) had a large home range concluding that aquatic turtles have larger home range than semiaquatic turtles; and *K. integrum*, considered a semiaquatic species, has the third largest home range in the family Kinosternidae (considering 95% KDE; Table 4). However, it is important to note that the information concerning home range in Kinosternidae is still scarce, as there is only information of home range for nine of the 25 species. Furthermore, most studies have estimated home range using the MCP method, which has been criticized due to its sensitivity to extreme outlying locations, and generally can cause an overestimation of the home range (Powell, 2000). In this study, the results of MPC include habitats in which individuals of *K. integrum* were never observed (e.g., crops, uncovered soil).

**Table 4:** Mean of home range of some species of family Kinosternidae (including *Kinosternon integrum*) calculated by: minimum convex polygon (▷), 50% KDE (●), and 95% KDE (○). Data expressed in scientific notation ^✧^ 10^−1^; ^✧✧^ 10^−2^; ^✧✧✧^ 10^−3^

Species		Home range (ha)				Source
		Females	Males	All	Range	
*Staurotypus salvini*				12^✧✧^		Legler and Vogt (2013)
*Kinosternon acutum*		1.6^✧✧^	9^✧✧✧^	1.2^✧✧^	2^✧✧✧^–3.1^✧✧^	Vogt, Daht, Espejel‐González, and López‐Luna (2000)
		2^✧✧^	1^✧✧^			Iverson and Vogt (2011)
*Sternotherus odoratus*	▷	4.9^✧✧^	2.4^✧✧^			Mahmoud (1969)
		9.4^✧^	1.7	1.2		Ernst (1986)
	▷	25.4	20.9	23.9		Belleau (2008)
	●			1.5	2.6^✧^–7.4	Rowe et al. (2009)
	○			2.8		“
	▷			6.6	62.2^✧✧^–22.1	Picard, Carrière, and Blouin‐Demers (2011)
	▷	8.2	11.6	9.9		Banning (2012)
	●	5.3	5.0			“
	○	1.0	0.9			“
	▷				1–5.2	Attum, Cutshall, Eberly, Day, and Tietjen (2013)
	▷	25.5	105.9	65.7	6.8–204.9	Bennett, Keevil, and Litzgus (2015)
*Sternotherus minor peltifer*	▷	5.2^✧^	5.2^✧^	51.8^✧✧^		Ennen and Scott (2013)
*Sternotherus depressus*	▷			8^✧✧✧^		Dodd, Enge, and Stuart (1988)
*Kinosternon subrubrum*	▷	4.8^✧✧^	5.2^✧✧^	5^✧✧^		Mahmoud (1969)
	▷	10.2	26.0	17.5		Cordero, Reeves, and Swarth (2012)
*Kinosternon flavescens*	▷	12.5^✧✧^	10.5^✧✧^			Mahmoud (1969)
***Kinosternon integrum***	▷	**48.6** ^✧✧^	**14.4** ^✧^	**97.7** ^✧✧^	**3** ^✧✧^ **– 149.7** ^✧^	**This study**
	●	**10.6** ^✧✧^	**19.8** ^✧✧^	**15.2** ^✧✧^	**6** ^✧✧^–**46.9** ^✧✧^	“
	○	**47.8** ^✧✧^	**83.7** ^✧✧^	**65.7** ^✧✧^	**2.8** ^✧✧^ **– 18.8** ^✧^	“
*Kinosternon scorpioides*	* *			4.5^✧✧^		Berry and Iverson (2011)

Species are mentioned according to Iverson, Le, and Ingram (2013).

Text in bold indicates the results of this paper

In turtles, intra‐ and interspecific variation on home range and patterns of movements of turtles has been reported (Slavenko et al., 2016). Home range and movements can be more influenced by extrinsic factors (i.e., weather, season, size of wetlands, environmental variation, and distribution of resources and food) than intrinsic factors (i.e., sex, size, and sexual maturity.) (Slavenko et al., 2016). The movements of *K. integrum* were influenced by extrinsic factors such as season and distance between microhabitats essential for survival (estivation sites and artificial ponds), but the distance traveled is consistent among years. The intrinsic factors such as size or sex did not affect the movement distance, which has been reported in other species (*K. subrubrum,* Bennett et al., 1970; *K. baurii,* Wygoda, 1979). Hence, identification of factors that promote the movements within a population could improve future conservations plans.

Most of the individuals of *K. integrum* in Tonatico presented low mobility and high fidelity for their aquatic habitat within the river (25.1% of the movements were <5 m). *K*. *integrum* exhibited both aquatic (within the river) and terrestrial movements (toward artificial ponds and estivation sites), which have been recorded in other species of Kinosternidae. According to Ennen and Scott (2008), and our field observations, some turtles did not show constant movements and remained in the same site for multiple days. Additionally, the movements of *K. integrum* were sudden and brief (especially long‐distance terrestrial movements). For example, in a range of three days, one male (M 5) did not move (0 m), and another male (M 20) moved 299 m (both during dry season). Previous research has suggested that turtles can migrate and later return to the same site with great accuracy (Morales‐Verdeja, 1991). This behavior was also observed in *K. integrum*, for example, one female (H 28) was estivating near the river, then migrated to an artificial pond (398.9 m), and next year returned at the same estivation site. Similarly, a male (H 5) was recaptured after 329 days (after 19 samplings) in the same pond in the river. These results strongly support the hypothesis that most individuals of *K. integrum* show a high fidelity for their habitats, and similar patterns have been observed in other Kinosternids (Tables 4 and 5). The high fidelity presented in turtles has been related to the availability of resources in the environment. The low mobility of *K. integrum* suggests that the Zapote River offers optimal environmental conditions to obtain resources locally that adequately satisfy the different energy demands of individuals. Interestingly, a similar situation has also been suggested by previous studies (Milam & Melvin, 2001).

**Table 5:** Mean of distance of the movements of some species of family Kinosternidae (including *Kinosternon integrum*). The estimation of the movements by ^✢^ linear home range, ^◼^ total daily distance movement, ^★^ mean distance, ^✸^minimum movement distance, ^◻^ distance between captures

Species		Movement (m)				Source
		Females	Males	All	Range	
*Kinosternon leucostomum*	^✸^			296^a^	0–600	Morales‐Verdeja and Vogt (1997)
	^✸^			277^b^		
	^✢^			105.8		
*Sternotherus odoratus*	^★^			320	0–1175	Smar and Chambers (2005)
	^◼^			27	0–314	Rowe et al. (2009)
	^◻^	44.7	67.57	44.5	1.8–525.5	Mahmoud (1969)
	^◻^	89.5	117.3	93.6		Ernst (1986)
*Sternotherus carinatus*	^◻^	17.4	38.6		4.5–93.8	Mahmoud (1969)
*Sternotherus minor peltifer*	^✢^			341		Ennen and Scott (2013)
*Sternotherus depressus*		19.2	31.2			Dodd et al. (1988)
*Kinosternon baurii*	^★^			12	1.1–48.8	Wygoda (1979)
*Kinosternon subrubrum*	^◻^	61.9	52.2		0.6–408	Mahmoud (1969)
	^◻^				1–600	Bennett et al. (1970)
	^◻^	82.9^a^	127.3^a^	119.2^a^	36.3–581.3	Harden et al. (2009)
				106^a^	40–198	Steen, Sterrett, Miller, and Smith (2007)
*Kinosternon flavescens*	^◻^	213.5	651.5	213	3.3–435.2	Mahmoud (1969)
*Kinosternon sonoriense*	^✸^			19^a^	1.0–79	Ligon and Stone (2003)
	^◻^	57.6	127.1		0–720	Hall and Steidl (2007)
	^✸^	172.6	75.6		0–1540	Hensley, Jones, Maxwell, Adams, and Nedella (2010)
***Kinosternon integrum***	^◻^	**56.1**	**47.93**	**51.44**	**0**–**662.4**	**This study**
*Kinosternon scorpioides*	^★^			68.27	?−380	Forero‐Medina and Castaño‐Mora (2011)

a Movements to estivation sites.

b Movements to nesting sites. Species are mentioned according to Iverson et al. (2013). Text in bold indicates the results of this paper.

Terrestrial movements have been recorded in other species within the *Kinosternon* genus, and long‐distance movements are rare. Nevertheless, some organisms within populations can perform large‐scale movements within the same habitat or among habitats (Bowne, 2008; Gibbons, Greene, & Congdon, 1983; Hall & Steidl, 2007; Ligon & Stone, 2003; Pittman & Dorcas, 2009; Smar & Chambers, 2005; Stone, 2001). In environments where the water is scarce, the number of terrestrial movements by turtles should be relatively high as turtles may need to move to ephemeral pools when they become available and to more permanent pools or to estivation sites when ephemeral pools dry (Stone, 2001). Herein, we present a similar result, because during the samplings we found that turtles migrate temporally to other sites. The terrestrial movements performed by *K. integrum* were carried out between river and estivation sites, and between river and artificial ponds. These strategies are present to avoid periods of droughts presented during the dry season (November–May) in the habitat of this population of *K. integrum*. The largest terrestrial movements carried out between river and artificial pond were recorded during the onset of the rainy season. This indicates that some individuals of *K. integrum* use permanent ponds of the river as a refuge during the dry season, but during the beginning of the rainy season migrate to temporal ponds (artificial ponds).

Roe and Georges (2007) revealed that the movements between water bodies were not species‐specific and some species of turtles use more than one wetland, normally traveling between two and three bodies of water. In the study site, it is common that some individuals of *K. integrum* perform seasonal migrations between river and artificial ponds; this ability to move between water bodies allows them to have a better survival condition and body condition, as the permanent pools can offer different resources and benefits, while others have dried (Roe & Georges, 2007, 2008). Because of this, the maintenance of landscape connectivity can be an important issue in the management of habitats adjacent to water bodies (ground safety zone). The conservation of the whole area is very important, as these environmental changes can be detrimental on the natural history of these organisms. Anthropogenic barriers (i.e., crops, roads) have a profound effect upon the survival of the turtles, mainly on those that perform overland movements (Myfsud & Myfsud, 2008; Pittman & Dorcas, 2009). It has been documented that in areas where disturbances occur in their habitat, turtles move greater distances (Plummer & Mills, 2008). Hence, conservation plans should include actions to maintain natural characteristics of the rivers and promote land corridors, in order to ensure that turtle populations are not negatively impacted.

*Kinosternon integrum* living in the Zapote River in Tonatico remained active throughout year. Although their activity decreased considerably during the colder months of the year (December–February), activity did not stop completely which differs from what was reported by Macip‐Ríos et al. (2009) in a nearby population of *K. integrum*. However, these differences may be due to the type of environment the turtles occupy. For this study, permanent ponds formed during the dry season along the river, which provided an adequate environment for some individuals to remain active, in comparison with artificial and temporal ponds in study area of Macip‐Ríos et al. (2009).

The present study increases our knowledge of turtle home range size, habitat use, and movement patterns of *K. integrum* turtles. The methodology utilized in this study examined 37 turtles using radiotelemetry; most recent studies have utilized under 25 individuals (Ennen & Scott, 2013; Ghaffari et al., 2014; Kapfer, Muñoz, Groves, & Kirk, 2013). Furthermore, the current study was conducted for 3.5 years, whereas previous studies have only monitored movements for 1 calendar year or less (Belleau, 2008; Rowe et al., 2009). Lastly, this study provides a thorough examination of the home range and movement patterns of *K. integrum* and provides invaluable data that can be used in future conservation practices and protection plans.

## CONFLICT OF INTEREST

None declared.
